# Catching the Elusive *Phytophthora*: A Review of Methods and Applications for Pathogen Detection and Identification Across Agricultural, Horticultural, Forestry and Ornamental Settings

**DOI:** 10.3390/biotech15010017

**Published:** 2026-02-09

**Authors:** Viola Papini, Alessandra Benigno, Domenico Rizzo, Salvatore Moricca

**Affiliations:** 1 Department of Agricultural, Food, Environmental and Forestry Science and Technology (DAGRI), Plant Pathology and Entomology Section, University of Florence, 50144 Florence, Italy; alessandra.benigno@unifi.it; 2 Laboratory of Phytopathological Diagnostics and Molecular Biology, Plant Protection Service of Tuscany, Via Ciliegiole 99, 51100 Pistoia, Italy; domenico.rizzo@regione.toscana.it

**Keywords:** phytopathogenic oomycetes, conventional identification, molecular detection assays, immunological techniques, in situ detection, VOC diagnostics, quarantine

## Abstract

Species of the genus *Phytophthora* are among the most detrimental plant pathogens globally, representing a significant threat to global agriculture, horticulture, and forestry. These zoosporic oomycetes have historically caused devastating outbreaks, including, just to mention a few, late blight of potato in Ireland; jarrah dieback of eucalyptus in Western Australia; ink disease of chestnut in Europe; sudden oak death and sudden larch death of coast live oak and tanoak in the Western US, and of Japanese larch in the UK. The environmental and ecological impacts of the diseases they cause result in significant economic costs that often have social repercussions. With the acceleration of globalization, enhancing the movement of plant material, in particular with the global live plant trade, the spread of *Phytophthora* to new, uncontaminated territories has intensified. Nurseries play a key role in the movement of these pathogens, the trade of contaminated stocks representing their major dissemination route. However valuable, conventional detection techniques, including baiting and direct isolation, are too slow and labour-intensive to meet current diagnostic requirements, particularly given the huge volumes of plants traded globally. This problem becomes even more acute when large volumes of potentially infectious plant material need to be processed in a short time frame, as it is often necessary to provide accurate and timely responses to interested parties. Early and precise detection is thus vital to avert outbreaks and mitigate long-term consequences. This review evaluates and contrasts the efficacy of novel detection methods against traditional approaches, emphasizing their significance in managing the escalating threat posed by *Phytophthora* spp. worldwide. Despite technological advances, critical challenges remain that limit the reliability and large-scale adoption of new diagnostic methods. Research still needs to bridge the gap between the laboratory and the field in terms of accuracy, sensitivity and diagnostic costs. Recent innovations focus on sensor technology and point-of-care (POC) devices for faster, more sensitive, and low-cost specific detection of *Phytophthora* spp. in plant matrices, water and soil. Enhancing diagnostic capabilities through these tools is crucial for protecting agricultural productivity, local economies, and natural ecosystems.

## 1. Introduction

A broad spectrum of host plants, encompassing taxonomically unrelated botanical families, consistently face an unremitting threat annually from oomycetes (kingdom Stramenopila) of the genus *Phytophthora* [1,2]. Members of this genus represent some of the most consequential threats to agricultural and forest ecosystems, inducing devastating diseases across continents on hundreds of plant species. These eukaryotic fungus-like organisms produce both sexual and asexual spores, which play crucial roles in their evolution and virulence. Each year, more members of this genus become part of this increasingly serious threat, which includes both native species exhibiting a recrudescence of the diseases they cause, and introduced species, the effects of which can be devastating due to a lack of co-evolution with the new hosts [3,4,5]. *Phytophthora* spp. are thus accountable for extensive tree mortality in various regions of the world, numerous well-documented instances of severe mortality and progressive dieback underscoring their impact, such as *Phytophthora alni* and its hybrids to *Alnus* spp. in natural formations and plantations [6]; *Phytophthora kernoviae* causing bleeding stem lesions on various forest trees [7]; *Phytophthora acerina* devastating *Acer pseudoplatanus* plantations in northern Italy [8]; *Phytophthora ramorum* [9] causing ‘sudden oak death’ in the Western US and ‘sudden larch death’ on Japanese larch in the UK; jarrah dieback associated with *Phytophthora cinnamomi* in southwestern Australia [10,11] and ink disease, one of the most destructive diseases affecting *Castanea sativa* Mill., associated with *P. cinnamomi* and *P.×cambivora* and with a complex of other *Phytophthora* spp. [12]. In these cases, *Phytophthora* spp. were identified as the primary drivers of the disease. In other situations, these pathogens act as one of the contributing factors to dieback, such as in the syndrome of oak decline, where *Phytophthora* appears to be a key contributor to the decline and mortality of oak trees [13,14]. Beyond forestry, *Phytophthora* spp. that cause significant economic losses in a wide range of crops, besides the notorious potato blight associated with the pathogen *Phytophthora infestans* [15], include, for example, *Phytophthora fragariae* [16,17], *Phytophthora capsici* [18], *Phytophthora nicotianae* [19], and *Phytophthora cryptogea* [20], which are responsible for significant crop damage and reduced yields. Globally, over 66% of root diseases and more than 90% of collar root issues in woody plants are attributed to *Phytophthora* spp. [21]. Nonetheless, in many instances, abiotic factors or secondary pathogens—rather than primary *Phytophthora* pathogens—are regarded as the principal causes of disease [21,22]. Concerns have also been raised about the occurrence of *Phytophthora* spp. in nursery environments, due to the risk of rapid spread if an infected plant were to be introduced into uncontaminated fields, forests, or urban green spaces [23,24]. This issue is particularly alarming given its direct impact on agricultural productivity, forest health, and urban greenery, alongside the potential for horticultural nurseries to act as significant hubs for the distribution of plant material through trade. Such activities facilitate the dissemination of *Phytophthora* spp. via contaminated green waste [25] and infected plants, which may then disseminate pathogens and pose a threat to biodiversity. For instance, *Phytophthora boodjera* has been detected in vegetation surrounding nursery production zones, inflicting disease losses in eucalypt nurseries [26]. Also *P. ramorum* was initially reported in North America in nurseries [27,28] before spreading with devastating outbreaks in natural forests of oak species, mainly on coast live oak (*Quercus agrifolia*), and the related tanoak (*Notholithocarpus densiflorus*) [29,30]. Furthermore, surveys of nursery stocks across Europe have identified a diverse array of *Phytophthora* spp.—more than twenty—found in forest trees, ornamentals, and irrigation systems [31]. The development of rapid and accurate diagnostic methods is crucial for several reasons: to enable early intervention (e.g., eradication); to implement appropriate management strategies, including preventive measures like checks at ports of entry (e.g., ports, airports and customs) and sensitive sites (e.g., food storage facilities, timber storage yards and nurseries); to optimise monitoring across the territory, to prevent the further spread of these pathogens from initial foci to new areas; to inform legislators so that they can enact appropriate legislative measures (e.g., bans on the importation of certain foodstuffs and plant materials from specific areas and other quarantine measures). This review provides an overview of current diagnostic approaches for detecting *Phytophthora*, emphasizing the urgent need for fast and dependable tools to address the growing threat of these pathogens in agricultural and forestry systems [32,33]. To meet commercial demands, these tools must also deliver quick results [34]. Detecting pathogens like *Phytophthora* is challenging due to their often non-specific symptoms [35], which hinder early detection; the need for selective substrates to isolate them [34] can complicate the identification process; co-infections can obscure diagnosis; and some *Phytophthora* spp. can survive in latency in the soil for an undefined time without inducing symptoms on a host [36], making their detection even more difficult. A comprehensive diagnosis generally involves combining traditional diagnostic techniques with molecular methods, with the former often supplemented by the latter.

## 2. Traditional Detection Methods

An extensive description of the conventional detection methods for *Phytophthora* spp. is documented by Erwin and Ribeiro [1], which essentially includes: direct microscopic examination of diseased plant material; isolation via baiting from infected plant tissues, water, and soil; and the use of general or selective agar media. *Phytophthora* spp. are known to infect only healthy plant material [37], and the pathogen may be present even in the absence of apparent symptoms. These organisms are notoriously challenging to isolate from necrotic tissue, as this often harbors numerous secondary pathogens. Consequently, isolating *Phytophthora* from dead plant tissue represents a significant challenge. Nevertheless, the most effective sampling strategy generally involves collecting samples from slightly affected trees or soil samples taken from around trees exhibiting symptoms or that have already succumbed to the disease.

### 2.1. Baiting

**Figure 1:**
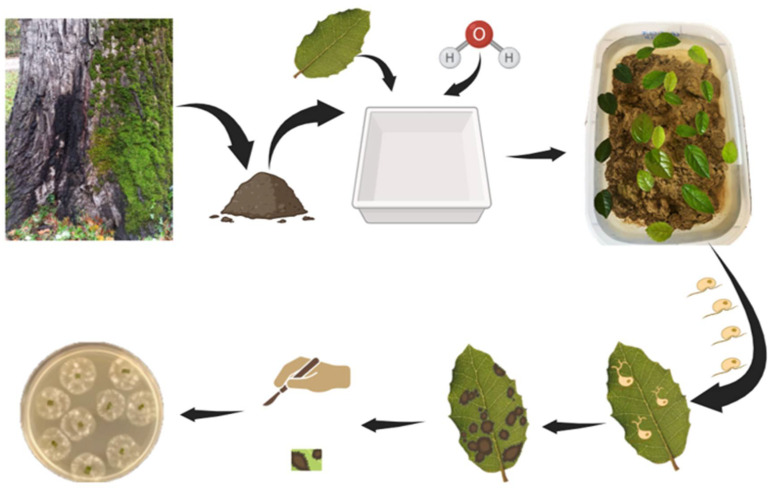
Diagrammatic representation of soil baiting procedure. Soil collected at the base of a plant exhibiting exudate symptoms is placed in a container partially filled with sterile water. After an incubation period of 24–48 h, leaf bait from a known susceptible host plant (e.g., *Viburnum tinus* L., *Quercus suber* L., or *Rosa chinensis* Jacq.) is floated on the surface of the water. If motile zoospores are present, encystment and infection will occur on the abaxial leaf surface, resulting in the development of necrotic lesions. Once foliar symptoms become evident, the leaves are rinsed in sterile water, then small leaf sections are excised from the lesion margin (between necrotic and healthy tissue) and transferred onto a selective agar medium for isolation.

**Figure 2:**
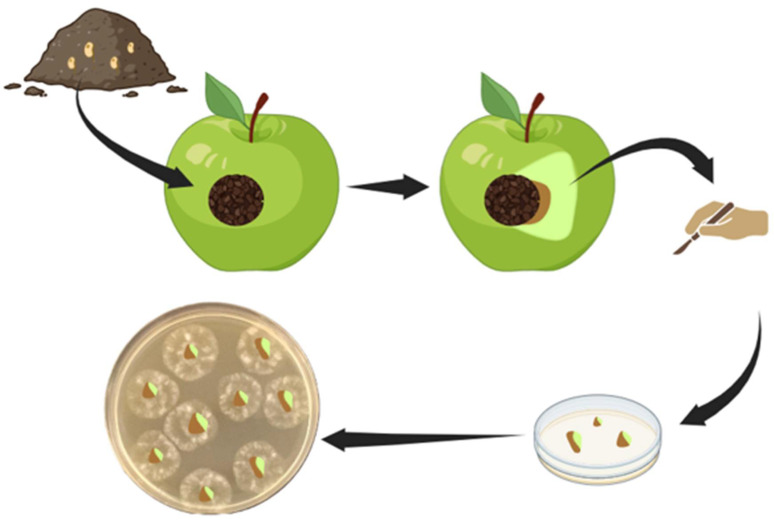
Diagrammatic representation of apple baiting procedure. Soil collected from the collar region of a symptomatic plant is applied to one or more holes (approximately 1 cm diameter and 2 cm deep) made on an apple with a sterilized scalpel. If the pathogen is present, brown rot will begin to develop in the pulp at the edge of the lesion where the inoculum (soil) was applied. A tissue sample encompassing both diseased and healthy tissue is then removed and placed on a selective medium to recover the pathogen.

**Figure 3:**
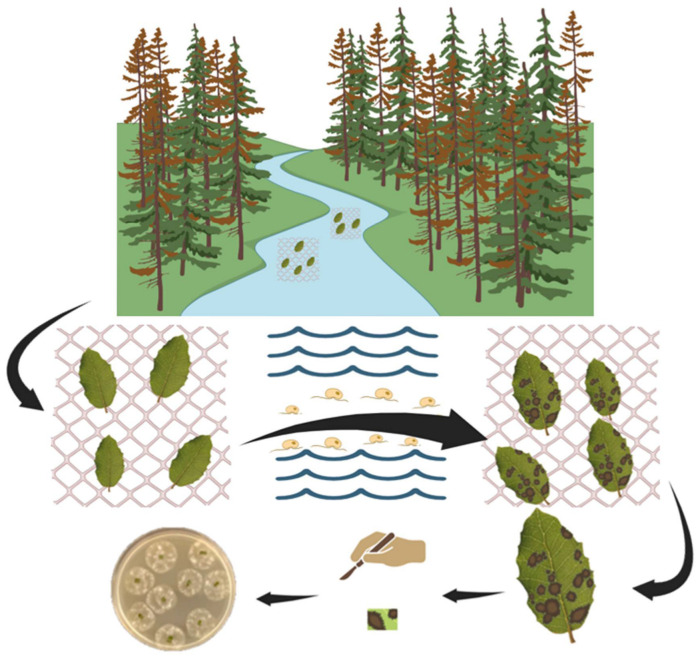
Diagrammatic representation of water baiting procedure. Baiting is conducted directly within a natural watercourse. Leaf baits enclosed within mesh traps are floated on the water’s surface and secured to the streambed with pickets or similar. Once symptoms appear on the leaves, the baits are retrieved, rinsed, and processed as described for the soil baiting method (Figure 1).

Baiting is a widely utilized method for the isolation of *Phytophthora* pathogens from several environmental matrices. This technique employs susceptible plant parts, which serve as living baits to attract motile zoospores released from sporangia. Zoospores are attracted to these baits owing to their negative geotropism and chemotactic behaviour, guiding their movement upwards, towards the bait [38]. Baiting effectiveness relies on several factors, such as the plant species used as bait, the duration, depth, and growth media used [39]. Common baits include leaf disks, cotyledons, fruits, and seedlings, with Rhododendron and oak leaves frequently utilized within forest ecosystems [1,40]. The appearance of a visible lesion on the bait tissue generally signifies a successful baiting procedure, as this lesion results from infection by *Phytophthora* spp. [41]. Subsequent culturing of the infected bait tissue on selective media allows for accurate and reliable identification of the causative species [1]. Several baiting techniques can be used for isolating *Phytophthora* from soil and water environments. Under laboratory conditions, baits are typically submerged in flooded soil trays to attract pathogenic zoospores [1,39,40], or apple baits are employed (Figure 1, Figure 2) [1,8,42,43]. Alternatively, baits can be directly incubated in rivers or irrigation canals (Figure 3). It is essential to note, however, that this process is semi-selective; it primarily detects species that produce zoospores, potentially overlooking microorganisms that lack swimming spores. This limitation may lead to a subsampling of the taxa actually present and an underestimation of species diversity [41]. Moreover, the specific conditions within laboratory environments can result in the failure to detect oospore populations, which serve as survival structures and likely comprise a significant proportion of *Phytophthora* inoculum in soil. These oospore structures may require distinct germination conditions not present in laboratory settings [44]. To address this challenge, a series of refined and advanced baiting protocols have been developed over time [45,46,47]. Despite its limitations, baiting remains a cost-effective, straightforward, and well-established method for pathogen detection. It is particularly useful for detecting *Phytophthora* in aquatic environments such as rivers, streams, and irrigation canals, where baits can be left submerged for extended periods to recover low pathogen levels. Nonetheless, in cases of negative results, it is advisable to perform multiple baiting attempts to confirm the absence of *Phytophthora* in the sample [37].

### 2.2. Isolation and Morphological Identification

The isolation of *Phytophthora* pathogens from various environmental samples, including plant tissue, soil, and water, is a critical step for accurate identification and subsequent research. A commonly employed method involves plating infected plant tissue onto various types of selective agar that contain both antibacterial and antifungal agents (Table 1). This selective medium facilitates the growth of *Phytophthora* while inhibiting the proliferation of other, unwanted microorganisms, thus enabling identification through its distinctive morphological characteristics [48,49]. Nonetheless, several challenges are associated with the isolation process. For example, the difficulty of *Phytophthora* to grow on selective agar is not always well understood but may result from antagonistic interactions with other microorganisms present in the tissue sample [50]; growth inhibition by plant phenolics [49]; or suppression of oospore or chlamydospore germination [48,51]. These factors can complicate the accurate detection of *Phytophthora* taxa. In soil and water samples, the use of selective media is essential to overcome the challenges posed by the overwhelming presence of contaminant microorganisms. Basic media, such as carrot agar (CA), potato dextrose agar (PDA), maltose dextrose agar (MEA), V8 medium, cornmeal agar (CMA), and water agar (WA) [37], are often insufficient for isolating *Phytophthora* from host tissues or baiting procedures; however, the application of selective media has considerably increased the success rate of pathogen isolation. Nonselective media are unsuitable due to competition from other microorganisms. This is particularly so if isolation depends upon the emergence of dormant *Phytophthora* propagules such as oospores and chlamydospores [22]. A low nutrient agar medium, such as cornmeal agar, is frequently used as a basal medium for *Phytophthora* isolation together with antimicrobial compounds that are inhibitory to a wide range of undesired, nontarget fungi or bacteria. Examples include 3-P medium, cornmeal agar with the addition of pimaricin, penicillin, polymyxin B [52]; P_10_VP medium, cornmeal agar with the addition of pimaricin, vancomycin and PCNB [53]; P_10_ARP medium, cornmeal agar with pimaricin, ampicillin, rifampicin and PCNB [54]; and hymexazol-amended medium, basic medium with added benomyl, PCNB, nystatin, ampicillin, rifampicin and hymexazol [55]. The hymexazol can be added to other selective media. However, the addition of hymexazol, which suppresses many *Pythium* and *Mortierella* species, can also be toxic to some *Phytophthora* spp. and may produce unrepresentative results [1]. NARPH medium with nystatin 22.72 ppm, ampicillin 100 ppm, rifampicin 10 ppm, pentachloronitrobenzene (PCNB) 100 ppm, and hymexazol 50 ppm [49] has been widely used. The carcinogenic properties of PCNB [56], which was a component of the original formulation, mean that this compound should not be used, and it has been eliminated by many researchers, like Simamora et al. [26], who use NARH (nystatin, ampicillin sodium, rifampicin, and hymexazol. The recipes for these selective media are based on commercially prepared basic media, as seen earlier with the CMA. Additionally, V8 agar can also serve as a basal medium, such as PARPNH-V8 agar medium [57]. Worth noting in the isolation of *Phytophthora* is the use of synthetic mucor agar (SMA), consisting of dextrose, asparagine, KH_2_PO_4_, MgSO_4_, thiamine HCL, agar, and distilled water. It is found in the literature in various modified forms and with the addition of antibiotics and antifungals as needed [58,59].

**Figure 4:**
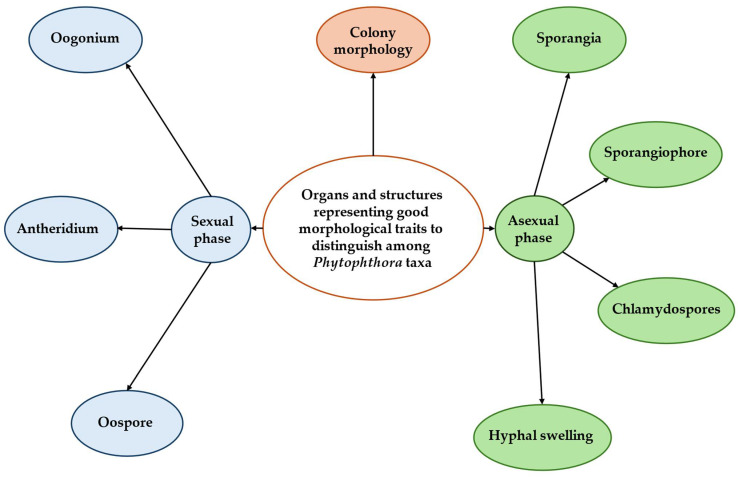
Useful morphological characters for identifying *Phytophthora* spp.

**Figure 5:**
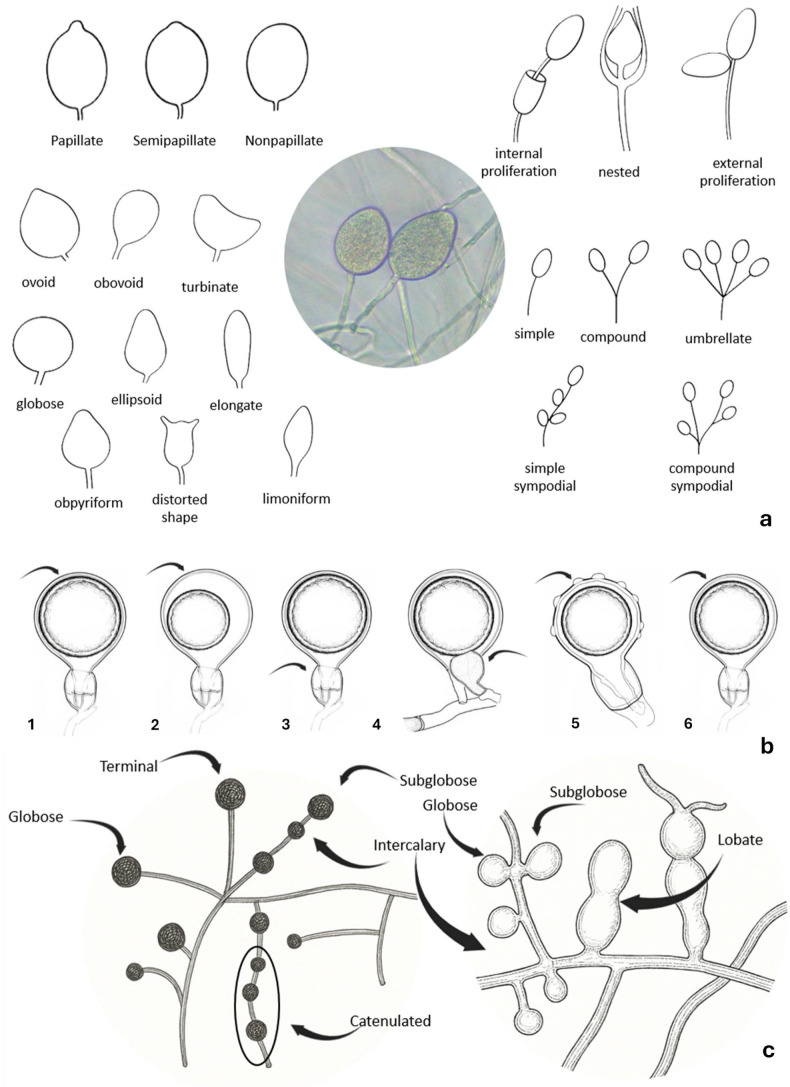
Some key morphological characters for identifying *Phytophthora* spp.: (**a**) asexual structures showing variation in sporangial morphology, such as degrees of papillation (papillate, semipapillate and non-papillate), types of proliferation (internal, external and nested) and shape diversity; (**b**) sexual structures showing the following cases: 1 and 2—oospore shape (plerotic and aplerotic, respectively); 3 and 4—antheridium shape (paragynous and amphigynous, respectively); 5 and 6—oospore wall (ornamented and smooth, respectively); (**c**) hyphal swellings and chlamydospores showing variation in position (terminal or intercalary) and morphology (globose, subglobose, lobate, or catenulated).

Identifying *Phytophthora* spp. requires examining traits from both the asexual and sexual phases, along with colony appearance (Figure 4, Figure 5). Important asexual features include sporangial papillation and caducity. The length of the pedicel remaining on caducous sporangia is another good taxonomic character. Sporangia may show internal or external proliferation, such as being extended or nested. Sporangiophores can be simple (unbranched) or branched; branched forms are classified as umbellate, simple sympodial, or compound sympodial. Hyphal swellings and chlamydospores, when present, may occur at terminal or intercalary positions and vary in shape and distribution. Although sporangial shape varies, it is often less useful for diagnosis. In the sexual stage, key features include reproductive mode (homothallic and heterothallic), gametangial morphology, and oospore type (plerotic or aplerotic). Colony morphology, including growth pattern, texture, compactness, surface topography, and growth rate on selective media, offers additional diagnostic clues [4,65].

Despite their utility, traditional isolation methods based on morphological and cultural criteria are time-consuming, require specialized microbiological/mycological expertise, and may not always yield conclusive results. These methods are often insufficiently sensitive to detect *Phytophthora* at a presymptomatic stage, and it is widely acknowledged that failure to detect *Phytophthora* via baiting techniques does not necessarily indicate its absence [1]. *Phytophthora* exhibits seasonal activity and fluctuating populations, which can rapidly shift from undetectable levels to high inoculum densities, particularly in natural ecosystems [66,67]. Therefore, the absence of *Phytophthora* in an environment should be interpreted with caution. One significant advantage of successful isolation is obtaining objective proof of *Phytophthora* presence, which also enables access to pure cultures for further characterization and research. Establishing key culture collections enhances subsequent analyses and contributes valuable data to our understanding of this group of pathogens. In complex natural environments, such as plant tissues, pathogenic microorganisms constitute a minority among a diverse range of agents that rapidly colonize the infected host. Despite the employment of selective media, isolating pathogens can be challenging due to the prevalence of undesired fungi or bacteria that grow more rapidly on the isolation plates. Therefore, serological or molecular analyses are generally preferred for diagnosis.

## 3. Immunodetection Methods

**Figure 6:**
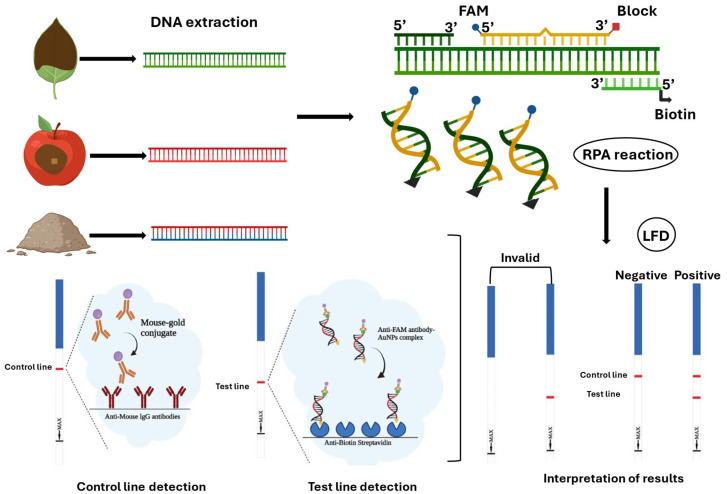
Illustration of the RPA-LFD test used to detect *Phytophthora cinnamomi* (modified from Chen et al. [85]).

Serological and immunological techniques have proven highly effective in detecting *Phytophthora* spp. by plant tissues, soil and water samples. These methods can be adapted to provide simpler and faster testing options suitable not only for laboratory analysis but also for field applications [68]. Both polyclonal and monoclonal antibodies targeting *Phytophthora* spp. are commercially available, enabling the development of specific detection assays [69]. Among these, the enzyme-linked immunosorbent assay (ELISA) remains a widely adopted tool. This biochemical diagnostic approach is based on antibodies that recognize specific antigens of plant pathogens, producing a colorimetric change upon binding. Commercial ELISA kits for *Phytophthora* detection are available in various formats, including high-throughput applications utilizing multi-well plates. These kits have demonstrated efficacy in identifying *P. cinnamomi* in azalea roots as early as one week post-inoculation [70], *Phytophthora fragariae* var. *rubi* in raspberry roots within four days [71], and *Phytophthora ramorum* [72]. Lateral Flow Devices (LFDs), also known as ImmunoStrips, provide point-of-care diagnostic solutions. An LFD is an easy-to-operate diagnostic device employing lateral flow immunoassay technology (LFIA), enabling rapid detection of specific antigens across various sample types without the necessity for sophisticated equipment. Using commercially available LFDs, *Phytophthora ramorum* and *P. kernoviae* have been detected in multiple oak species. The tests are straightforward, yielding results within 3–5 min, thus validating their utility. Comparative analyses with laboratory methods such as isolation and real-time PCR revealed sensitivities of 87.6% and specificities of 82.9%, with an overall efficiency of 95.6%, outperforming visual symptom assessments, which ranged between 20% and 30% for *P. ramorum* and *P. kernoviae*. This proves the importance of LFD tests as effective diagnostic tools during inspections at sensitive sites such as ports, airports, customs, nurseries, etc., and as preliminary field screening to identify samples that require detailed laboratory analysis for species identification [69]. Relative studies indicated that immunological methods, including ELISA and LFD, possess higher sensitivity for *P. ramorum* detection compared to species-specific PCR, thus serving effectively as pre-screening tools [72]. In aquatic environments, zoospore trapping immunoassays (ZTI) have demonstrated superior sensitivity in detecting *Pythium* and *Phytophthora* spp. compared to traditional methods, while immunodiagnostic dipsticks have proven effective for in situ assessments of infected root zones [73]. These immunological techniques are thus valuable tools for rapid and sensitive pathogen detection across diverse contexts. Pettitt [74] characterized the zoospore-trapping immunoassay as a suitable methodology for water monitoring aimed at detecting and identifying *Phytophthora* spp., while dipsticks, LFDs and ELISA were classified as moderately to highly effective monitoring approaches. The advantages associated with these methods, similar to those based on the ELISA, include the capacity to deliver prompt results without necessitating pathogen isolation. They have successfully detected *Phytophthora* spp. in various sample types, including plant foliage, woody tissue, roots, soil and irrigation water [75]. In certain instances, these assays serve as pre-screening tools to minimize the number of samples requiring subsequent, more costly, and time-consuming confirmatory testing procedures, thereby optimizing large-volume sample studies [69,76]. A notable example is the approval of a high-throughput ELISA protocol as a primary screening method within national surveys for *P. ramorum* [77]. Furthermore, LFDs offer additional advantages by facilitating field-based diagnostics without the need for specialized laboratory infrastructure. They are user-friendly, portable, and capable of providing results within ten minutes or less, thus supporting immediate decision-making in disease management [78,79]. Conversely, these assays exhibit limitations, as positive results frequently require confirmation via PCR or isolation techniques [72,80]. Cross-reactivity, particularly among closely related oomycetes such as *Phytium* spp. and *Peronospora*, is also documented, potentially leading to false positives and misdiagnoses [81,82,83]. Recent advancements in plant pathology involve the integration of serological and molecular amplification methods to enhance the sensitivity, specificity and rapidity of pathogen detection, especially concerning *Phytophthora* spp. For instance, Dai et al. [84] developed a recombinase polymerase amplification coupled with lateral flow dipstick (RPA-LFD) assay for the swift detection of *Phytophthora sojae*, achieving a detection limit of 10 picograms of genomic DNA. This assay detected *P. sojae* in 55.4% of samples, surpassing other methods such as loop-mediated isothermal amplification (54.6%), conventional PCR (46.9%) and leaf-disc baiting (38.5–40.0%). Similarly, an RPA-LFD assay targeting the Pcinn13739 gene was designed for the detection of *Phytophthora cinnamomi* (Figure 6) [85]. This method demonstrated a detection limit of 10 pg.µL^−1^ of genomic DNA and exhibited greater sensitivity than traditional PCR diagnostics [85].

Furthermore, the portability of devices such as lateral flow devices (LFDs) offers significant advantages for field applications, as has been demonstrated in the diagnosis of other pathogens in the field [86,87,88]. An illustrative example is an on-site rapid detection system for *P. ramorum*, which employs loop-mediated isothermal amplification (LAMP) in conjunction with visualization on a lateral flow device (LFD), developed by Tomlinson et al. [89].

## 4. Molecular Detection Methods

### 4.1. Polymerase Chain Reaction (PCR)

Recently, DNA-based methodologies have emerged as the preferred approaches for pathogen detection due to their high specificity and robustness [90]. Techniques such as Polymerase Chain Reaction (PCR) and its variants are undeniably the most popular and extensively researched methods of nucleic acid amplification. Furthermore, advances in PCR amplification technology have further improved the sensitivity and reliability of these techniques [91,92]. Consequently, PCR-based methodologies have been widely employed in the detection of *Phytophthora* spp., demonstrating high levels of specificity and sensitivity. Conventional PCR amplifies target DNA using specific primers, thermostable polymerase, nucleotides, and thermal cycling [93]. Importantly, the assay specificity depends on primer design targeting unique genomic regions [94], with amplification typically confirmed by agarose gel electrophoresis. Over the years, researchers have developed PCR assays targeting various genomic regions, such as internal transcribed spacers (ITS) and intergenic spacer (IGS) regions of ribosomal DNA (rDNA) [95,96,97]. These approaches allow detection at the genus level and often enable discrimination among species. For example, Silvar et al. [97] developed three PCR primers, CAPFW, CAPRV1, and CAPRV2, specific for *P. capsici*; both primer sets, CAPFW/CAPRV1 and CAPFW/CAPRV2, demonstrated in conventional PCR a limit of detection of 5 pg, whereas in nested PCR, the detection limit for both was 0.5 fg. Despite that, the CAPFW/CAPRV2 set in conventional PCR was used to detect *P. capsici* DNA in inoculated plants. Detection occurred as early as 8 h post-inoculation in stem samples from infected but still symptomless plants. Similarly, primers targeting the ITS region have been demonstrated to detect *P. infestans* with high specificity [95]. As well as Judelson and Tooley [98], who employed highly repeated DNA sequences, developing sets of primers to detect the presence of *P. infestans*, reaching a detection limit of 10 fg and a good specificity. Another example is the work of Liew et al. [96], who sequenced the complete IGS 2 region of *P. medicaginis* and related species, and then developed oligonucleotide primers within the IGS2 of *P. medicaginis*. The resulting primers specifically amplified *P. medicaginis* DNA with high sensitivity, detecting as little as 4 ng of DNA, even in a host–pathogen DNA ratio of 1,000,000:1. This protocol, therefore, proved effective for identifying *Phytophthora* in infected plant tissues, providing valuable tools for disease diagnosis and management [96]. However, conventional PCR has certain limitations, including sensitivity to inhibitors often occurring in plant tissues [99,100], inability to distinguish viable from non-viable organisms [101] and a lack of quantification.

### 4.2. PCR-Based Variants

To overcome the challenges inherent in basic PCR, several modifications of the techniques have been developed.

#### 4.2.1. Multiplex PCR

Multiplex PCR enables simultaneous detection of multiple pathogens using different primer sets [102]. A notable example is the multiplex PCR, developed by Otsubo et al. [103] for quarantine control in Japan. This assay used primers specific for quarantined *Phytophthora* spp., as well as for *P. nicotianae*, the only non-quarantine *Phytophthora* spp., and as internal controls, for plants. Nonetheless, this technique may reduce sensitivity due to primer competition. Therefore, adjusting the primer concentration ratio is crucial to overcome amplification disparity resulting from primer competition [104].

#### 4.2.2. Nested PCR

Nested PCR increases sensitivity and specificity through two rounds of amplification using internal primers [105]. Despite its advantages, it is more labour-intensive and increases the risk of contamination [106,107]. For example, Schena et al. [108] developed a PCR-based “molecular toolbox”, or rather a nested PCR, with a first round using the genus-specific primers Yph1F-Yph2R [109], and a second with species-specific primers. This protocol showed high specificity and was sensitive enough to detect target species in infected leaves, infested soil, and water samples. Judelson and Tooley [98] showed that the developed set of primers, when used in nested PCR, achieved a detection limit of 0.1 fg of *P. infestans* DNA, whereas the detection limit for PCR was 10 fg. Ippolito et al. [110] made the same observation, emphasising that the nested PCR reached a detection limit of 1 fg μL^−1^, whereas the conventional PCR had a detection limit of 1 pg μL^−1^, thus enhancing sensitivity.

#### 4.2.3. Quantitative PCR

Quantitative PCR (qPCR) enhances diagnostic accuracy by enabling real-time quantification of DNA through the use of fluorescent detection systems, thereby eliminating the need for post-amplification gel electrophoresis [111,112]. This technique monitors DNA amplification using either non-specific DNA-binding dyes, such as SYBR Green I, or sequence-specific hybridization probes, including hydrolysis probes and molecular beacons, which provide higher specificity [111,113,114,115]. For example, Osawa et al. [116] developed a real-time PCR assay to estimate *P. infestans* population density and inoculum potential in upland soils. In a similar application, the population densities of *P. cactorum*, *P. cinnamomi* and *P. lateralis* in 128 soil samples from 32 kiwi orchards in China during 2017 and 2018 were quantified using multiplex real-time quantitative PCR based on the Ras-related protein gene Ypt1 [117]. Notably, Schenck et al. [118] developed a real-time PCR assay targeting the Ypt1 gene, achieving high sensitivity and specificity for detecting *Phytophthora lateralis* in plant tissues, with a detection threshold as low as 47 DNA copies.

#### 4.2.4. Droplet Digital PCR

**Figure 7:**
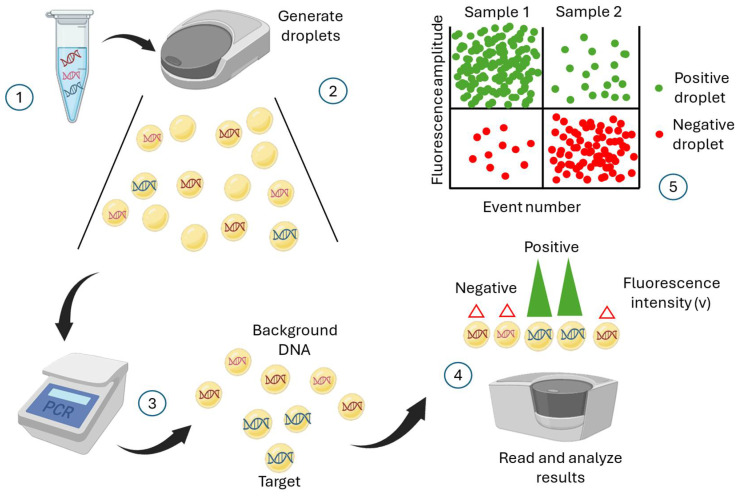
Schematic illustration of how droplet digital PCR (ddPCR) works. PCR amplification produces thousands of copies that can be detected and interpreted by a detection system. (**1**) In a typical ddPCR workflow, a single sample includes target and nonspecific sequences (DNA or RNA), real-time PCR primers and fluorescent-labelled probes, and standard real-time PCR master mixes. (**2**) A sample is partitioned into thousands of single-nanoliter droplets with the generation of water-in-oil emulsions. A proportion of droplets contain no template molecules, while others contain one or more targets. (**3**) The PCR is then performed to amplify the target sequence. (**4**) Target sequence droplets exhibit higher fluorescence intensity and are known to be positive droplets. Empty or no targets show low and negative fluorescent intensity. This fluorescence intensity versus time is plotted on a graph. Various methods can interpret the fluorescent intensity of droplets. The most popular of these is a fluorescent microscope or a droplet reader. (**5**) The acquired data is visualized in a graph by different software.

Beyond qPCR, droplet digital PCR (ddPCR) is utilized as a diagnostic tool for detecting and quantifying plant pathogens, providing advantages over traditional quantitative PCR (qPCR) (Figure 7). Studies have demonstrated ddPCR’s high sensitivity and precision in detecting *Phytophthora nicotianae* in tobacco plants and soil samples [119,120]. The technique exhibits greater tolerance to PCR inhibitors and enhanced accuracy at low pathogen concentrations [119,120]. Ristaino et al. [121] developed a loop-mediated isothermal amplification (LAMP) assay for *P. infestans* and compared this method to conventional PCR, real-time LAMP, and droplet digital PCR for detection purposes. Droplet digital PCR demonstrated the lowest detection threshold (100 fg/µL), compared to conventional PCR with 10 pg/µL and 584 fg/µL for SYBR Green LAMP read on the mReader. These findings underscore the potential of ddPCR as a robust and reliable method for early diagnosis of plant pathogens in complex environmental samples, thereby informing disease management strategies.

Overall, PCR-based methodologies are extremely versatile and reliable tools for detecting plant pathogens, including *Phytophthora* spp. Technological improvements have continuously refined their sensitivity, specificity and applicability in different diagnostic contexts. These techniques are therefore a key reference point in molecular pathogen analysis and provide the basis for integration with more recent, complementary diagnostic approaches.

### 4.3. Isothermal Amplification Methods

**Figure 8:**
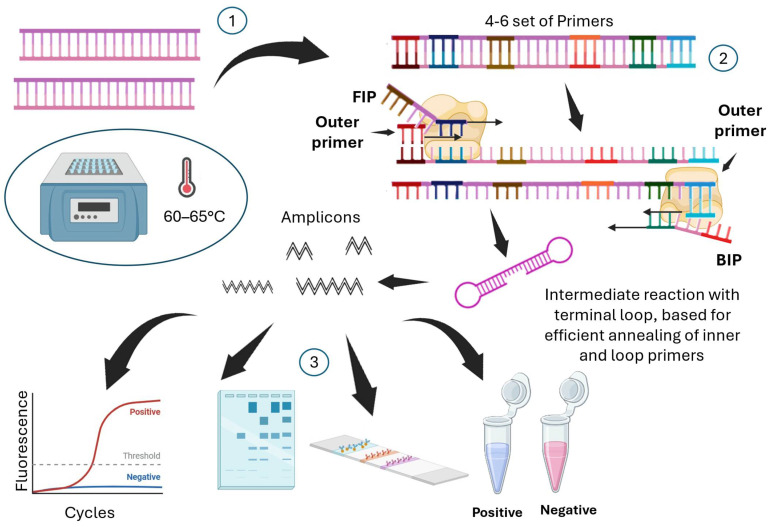
Diagrammatic representation of the Loop-Mediated Isothermal Amplification (LAMP) method. (**1**,**2**) The reaction employs a specific set of four to six primers that recognize six different regions of the target DNA. The amplification process starts with the inner primers (FIP and BIP), which anneal to the partially denatured template DNA and are extended by DNA polymerase. Then, the outer primers anneal to the same single-stranded DNA (ssDNA) template strand and are extended by the polymerase. This ultimately results in the formation of a characteristic reaction intermediate with terminal loops. This structure acts as the core intermediate for the annealing of inner and loop primers, driving the exponential phase of the reaction. As a result, there is a rapid build-up of a large quantity of amplicons of various sizes. (**3**) The DNA products can be detected using different methods, including real-time fluorescence analysis, gel electrophoresis visualization, colorimetric dyes, or lateral flow devices.

Conversely to PCR, which employs thermal cycling at three distinct temperatures to obtain DNA strand separation, primer annealing and target sequence extension, isothermal amplification enables nucleic acid amplification at a single temperature [122], obviating the need for energy-intensive and technologically complex thermal cycling processes. Due to these attributes, isothermal techniques are being adopted or considered promising for in-field point-of-care applications [123]. Among these, Loop-mediated isothermal amplification (LAMP) and Recombinase Polymerase Amplification (RPA) are the most commonly used methods for detecting *Phytophthora*. LAMP constitutes an alternative technique for amplifying pathogen templates in diagnostic applications. LAMP utilizes six gene regions for amplification. It involves the design of primers that anneal to distinct regions of the target, utilizing DNA polymerase with strand displacement activity to facilitate amplification at a constant temperature [122] (Figure 8). Notably, Khan et al. [124] demonstrated that a LAMP reaction conducted within 60 min at 65 °C could detect up to 1.28 × 10^−4^ ng μL^−1^ of pure genomic DNA from *P. infestans*. The LAMP assay exhibited no cross-reactivity with other *Phytophthora* spp., oomycetes, or fungal pathogens. Furthermore, the assay demonstrated a sensitivity capable of detecting DNA concentrations as low as 1.28 × 10^−4^ ng μL^−1^, which is tenfold higher than nested PCR (1.28 × 10^−3^ ng μL^−1^), one hundredfold higher than real-time PCR (1.28 × 10^−2^ ng μL^−1^), and 103-fold greater than conventional PCR (1.28 × 10^−1^ ng μL^−1^). A multiplex LAMP assay was developed in Japan to detect *P. ramorum*, *P. lateralis*, *P. kernoviae*, which are quarantined pathogens in this country, as well as the domestic *P. nicotianae* [125]. Furthermore, a study conducted in China involved the development of species-specific LAMP assays based on the tigA gene for the detection of the most widespread pathogen, *P. cactorum*, in soil from kiwi orchards [117]. Many other researchers have proposed LAMP assays as highly specific and innovative diagnostic methods, including Dai et al. [126], who employed a novel target gene, Pcinn100006, to evaluate the detection of *P. cinnamomi*.

RPA is a technique comparable to PCR but differs in that it utilizes enzymatic processes instead of thermal cycling for DNA strand separation and primer annealing [127]. The RPA reaction can be conducted within a temperature range of 30–42 °C, with an optimal temperature of 39 °C [128]. The simplicity of primer design provides a technical advantage over technologies such as LAMP. Similarly to LAMP, RPA exhibits relative insensitivity to common PCR inhibitors [129,130]. The sensitivity of RPA for rapid nucleic acid detection can be significantly enhanced through its integration with CRISPR-based technologies. Recently, this combined approach has been employed in the diagnosis of *Phytophthora* spp., such as *P. nicotianae* and *P. syringae* [131,132]. Notably, to enhance the detection of *P. pini*, Dai et al. [133] established both an RPA-LFD and an RPA-CRISPR/Cas12a assay. The former can be completed in approximately 30 min, while the latter requires 50 min. Regarding sensitivity, the lowest concentration detectable by RPA-LFD was 10 pg/μL, whereas RPA-CRISPR/Cas12a was capable of detecting *P. pini* at 1 pg/μL.

Recent advancements in isothermal amplification techniques have emerged following the application of helicase-dependent amplification (HDA), successfully utilized for detecting *Phytophthora* spp., particularly *P. kernoviae*, with the Ypt1 gene as the target [134]. This method, combined with on-chip hybridization and silver nanoparticle visualization, facilitates field-applicable detection with high sensitivity [134]. Similarly to LAMP, HDA amplicons can be detected through various methods, including gel electrophoresis, fluorescence, electrochemical detection and lateral flow assays. Despite its simplicity, HDA is susceptible to non-specific amplification, which is why thorough optimization and screening of primers are necessary to avoid false-positive and false-negative results [135].

### 4.4. DNA Micro-Macroarray-Based Methods

DNA arrays serve as instruments for gene expression profiling but can also be employed for the identification and differentiation of microorganisms [136,137]. Microarray and macroarray technologies have been engineered for the detection and identification of multiple *Phytophthora* spp. as well as other plant pathogens [138,139]. Sikora et al. [140] developed a padlock probe-based microarray method for the concurrent detection of various *Phytophthora* spp., employing a colorimetric readout, which discriminated nine *Phytophthora* spp. with species-specific resolution, while others were identified in groups. Wong and Smart [141] developed a chromogenic detection technique for a DNA macroarray system, offering an alternative to chemiluminescent detection for the identification of *Stemphylium solani* and *Phytophthora capsici*. Chen et al. [142] devised a membrane-based oligonucleotide array utilizing multiple DNA markers (ITS, cox1, and cox2-1 spacer) to detect and differentiate various *Phytophthora* spp. from environmental samples. Zhang et al. [138] designed a macroarray system employing oligonucleotides based on ITS sequences to detect predominant fungal and oomycete pathogens of solanaceous crops. These array-based methodologies provide multiplex detection capabilities for plant pathogens, including *Phytophthora* spp., across diverse matrices and specimen types. Consequently, DNA array technology has been successfully implemented for the concurrent detection of multiple microorganisms from various habitats, encompassing numerous *Phytophthora* spp.

### 4.5. Next Generation Sequencing (NGS)

Next-generation sequencing (NGS) techniques have emerged as powerful tools for the detection and identification of *Phytophthora* spp. in environmental samples. In comparison to conventional methods, NGS offers superior survey capacity, increased detection sensitivity and enhanced cost-efficiency [143,144]. This methodology provides invaluable guidance to stakeholders in crop protection, including regulatory agencies charged with plant health inspection and disease management [143]. In a recent study, *Phytophthora*-specific barcoded primers were employed to amplify the mitochondrial DNA spacer adenosine triphosphate synthase subunit 9–nicotinamide adenine dinucleotide dehydrogenase subunit 9 (ATP9-NAD9). Through this methodology, several *Phytophthora* spp. were identified using next-generation sequencing (NGS) and their identity was subsequently confirmed by species-specific quantitative polymerase chain reaction (qPCR) assays [143]. Multiple investigations have effectively utilized Next-Generation Sequencing (NGS) to assess the diversity of *Phytophthora* spp. in soil and water samples across various ecosystems [145,146]. For instance, amplicon pyrosequencing of environmental DNA (eDNA) has revealed an extensive diversity of *Phytophthora* spp., with as many as 37 phylotypes identified within a single study [145]. Català et al. [146] documented the detection of 13 *Phytophthora* spp. in soil samples, while water samples taken in the same locations yielded 35 species. This outcome highlights that water samples generally exhibit greater species diversity than soil samples. Additionally, the study emphasizes the methodological benefits of water sampling, since soil pre-processing is more laborious compared to the relatively expedient processing of water filters, which facilitates the preparation of amplicon libraries within a single day [146]. In a recent investigation, Landa et al. [147] employed high-throughput Illumina sequencing targeting the widely recognized Internal Transcribed Spacer 1 (ITS1) region of ribosomal RNA, comparing it with mitochondrial cytochrome c oxidase I (COI) gene markers to evaluate the diversity of *Phytophthora* in disturbed and undisturbed soils in Britain. Overall, the integration of various sampling methodologies with next-generation sequencing (NGS), validated by quantitative PCR (qPCR), enhances the capacity for surveys and the sensitivity of detection, while simultaneously decreasing manual labour and associated costs. These technological advancements are crucial in helping regulatory agencies identify potential entry points for *Phytophthora* spp. and improve phytosanitary procedures.

### 4.6. Principal Target Regions

Numerous coding (genes) and non-coding (intergenic) DNA regions have been utilized as preferred targets for the development of primers for pathogen typing and detection, and many of these have concerned strain identification of important *Phytophthora* spp. Because of the ease of amplifying the multicopy ribosomal gene regions, the ribosomal gene complex is commonly used for pathogen identification [148]. The most widely used nucleotide sequences are the Internal Transcribed Spacer (ITS) region, arranged within the Transcription Unit, between the nuclear Small and the nuclear Large Subunits rRNA gene; the non-transcribed Intergenic spacer (IGS), located downstream of the 25S (or 28S) gene and upstream of the 18S gene within the Repeat Unit; the 60S Ribosomal Protein L10, a conserved ribosomal protein located within the Large (60S) Subunit of the ribosome, specifically at the inter-subunit side, and flanking regions. Other preferred targets for primer design are the beta Tubulin, a microtubule constituent protein; enolase, an essential enzyme in glycolysis; heat shock protein 90, a cellular chaperone protein; the mitochondrial cox1 locus, encoding mitochondrial cytochrome oxidase; the mitochondrial cox2 locus, also encoding a mitochondrial cytochrome oxidase; the mitochondrial NADH Dehydrogenase Subunit 1, Mitochondrial NADH Dehydrogenase Subunit 1 and flanking regions; the Mitochondrial NADH Dehydrogenase Subunit 9, mitochondrial NADH Dehydrogenase Subunit 9, and flanking regions; mitochondrial ribosomal protein S10 and flanking regions; mitochondrial sec-independent transporter protein, mitochondrial sec-independent transporter protein (ymf16); TEF1, the translation elongation factor; and the TigA gene fusion, which involves the transcriptional fusion of genes encoding triose-phosphate isomerase and Glyceraldehyde-3-Phosphate Dehydrogenase (Table 2) (*Phytophthora* Database. Available online [149]).

## 5. VOCs

Recent research has focused on using volatile organic compounds (VOCs) as markers for identifying *Phytophthora* spp. P-ethylphenol has been recognized as a key VOC emitted by strawberries infected with *Phytophthora cactorum*, as shown in a study by Wang et al. [162]. Sherwood et al. [163] and Thompson et al. [164] proved that VOC profiles can distinguish different *Phytophthora* spp. grown in culture and can separate infected plants from healthy ones. The analytical approach combining headspace solid-phase microextraction (HS-SPME) with gas chromatography-mass spectrometry (GC-MS) has been fine-tuned to detect VOCs produced by *P. cinnamomi*, achieving high sensitivity and accuracy [165]. Additionally, VOC detection methods are effective for identifying infections that show no symptoms in nursery settings, with ambient volatile compounds providing better predictive results than those extracted directly from leaves [164]. Furthermore, innovative bioelectronic noses are emerging as a complementary approach for detecting and distinguishing volatile organic compounds (VOCs) associated with *Phytophthora*. These sensors typically feature an array of gas detection elements combined with signal processing tools, which provide high sensitivity to changes caused by VOC interactions across various detection modalities, such as electrical, optical, mechanical, or biological responses [166]. For instance, sensors based on single-walled carbon nanotubes (SWNTs) immobilized on field-effect transistors and functionalized with different single-strand DNA molecules (ssDNA) have shown the ability to selectively identify specific odours by detecting targeted volatile compounds [162]. It has been indicated by recent research that infected plants can be caused to release unique volatile organic compound (VOC) patterns by oomycetes, including certain pathogenic *Phytophthora* spp. that can be quickly identified using bioelectronic noses. This method enables the detection of pathogenic organisms and allows the differentiation between closely related species and genera. For example, it can distinguish between *Pythium intermedium* and *Phytophthora plurivora* [167]. Importantly, this technique significantly shortens diagnostic times bypassing the necessity for pathogen culturing and DNA extraction. These findings collectively suggest that VOC analysis constitutes a promising strategy for the early, rapid, and high-throughput detection of *Phytophthora* spp. across various plant materials and environmental contexts [163,165].

## 6. Operational Characteristics of Diagnostic Methods: A Comparative Analysis

**Figure 9:**
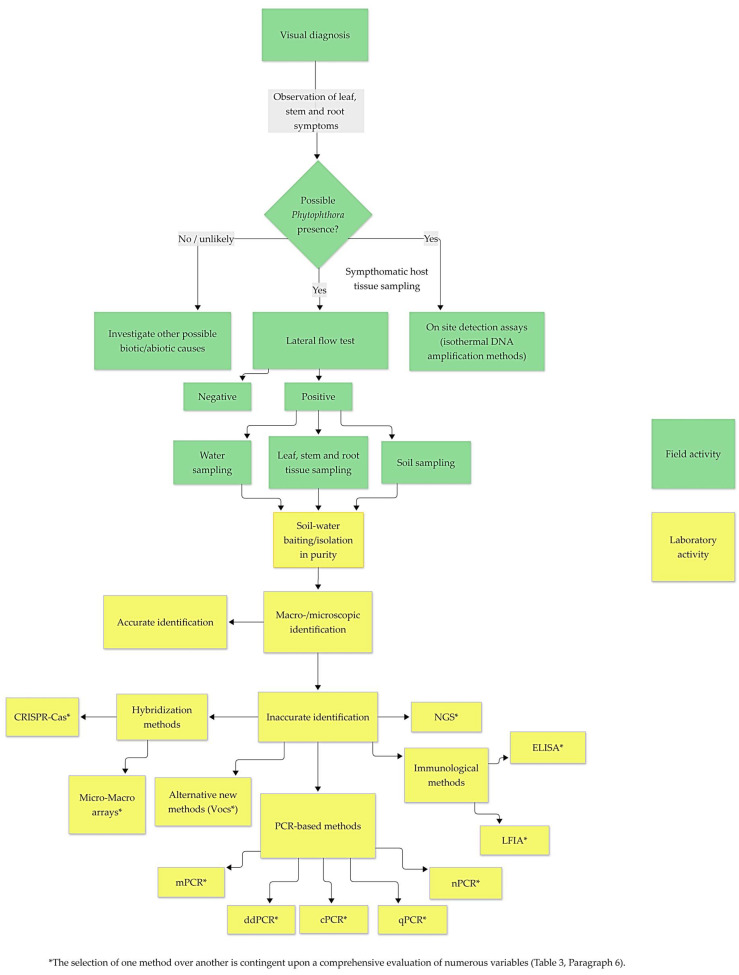
Flowchart for the detection and identification of *Phytophthora* spp., from visual field diagnosis to laboratory-based confirmation, with increasing levels of diagnostic complexity.

The diagnostic methods available today vary considerably in terms of characteristics and functions. As outlined in the diagnostic flow chart (Figure 9), the selection of these tests is multifaceted and contingent on numerous variables, as detailed in Table 3.

These factors influence their operational characteristics, which involve a balance between analytical performance, cost, and usefulness (Table 3). Traditional cultivation methods remain effective due to their affordability and the fact that they do not require advanced diagnostic tools. However, they demand significant mycological/microbiological expertise and are characterized by low throughput, slow turnaround times, and limited sensitivity, making them less suitable for rapid or large-scale diagnostics [168,169]. In contrast, high-sensitivity molecular tests like qPCR and ddPCR offer high specificity and accurate quantification but are costly and depend on substantial infrastructure [174,175]. Rapid immunoassays and isothermal amplification methods provide advantages in turnaround speed, affordability, and field deployment, making them suitable for decentralized or screening applications despite lower analytical performance [171,178,179,180,181,182,183,184]. In this context, genus-specific lateral flow devices for *Phytophthora* offer a simple, quick, equipment-free solution for field use, even by non-specialists like growers, making them ideal for primary screening to select samples for lab confirmation [69,72]. However, their diagnostic sensitivity and specificity are generally lower than PCR-based methods, and since most commercial devices target the genus level, identification usually remains at the *Phytophthora* spp. level and requires follow-up species-specific molecular tests if precise identification is needed [190]. Overall, these findings highlight that each diagnostic method has unique strengths and limitations, which should be carefully considered based on the specific diagnostic context and operational needs.

## 7. Protocol Validation

**Figure 10:**
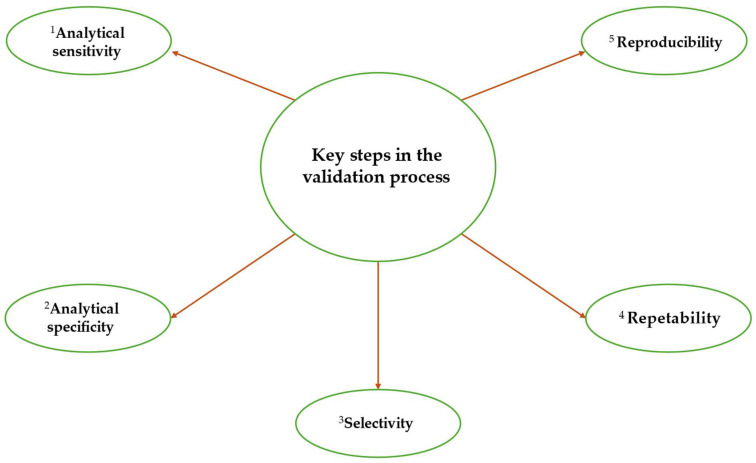
Essential checks for an accurate validation process of the molecular diagnosis of fungi and oomycetes (EPPO diagnostic standards) [191]. Performance criteria: ^1^ Analytical sensitivity: Conduct three experiments with serial dilutions in host plant tissue to identify the lowest amount of target DNA that remains detectable. ^2^ Analytical specificity: (i) Inclusivity: Analyze strains of the fungus or oomycete, considering genetic diversity, geographic distribution, and hosts. (ii) Exclusivity: Analyze relevant non-target fungi, especially those associated with the matrix. The nucleic acid concentration should be sufficiently high to maximize cross-reaction potential. ^3^ Selectivity: Assess whether variations in the matrix—such as age, conditions, plant part, cultivars, or soil type—impact test performance. ^4^ Repeatability: Test at least three replicates of samples with low target concentration, as determined by the sensitivity experiments. ^5^ Reproducibility: Similarly to repeatability, but performed by a different operator, on different days, and with different equipment where applicable.

Validation is a pillar of analytical quality and the reliability of diagnostic results. Diagnostic protocol validation is in fact a fundamental step in ensuring that the selected method is reliable, reproducible, and fit for purpose [191,192]. Regarding morphological and morphometric tests, it is recognised that these methods cannot be validated using the same rigorous criteria as other types of tests. In such cases, the process relies heavily on the experience and technical judgement of specialists. Expert judgement [191] usually involves consulting available documentation, such as identification keys, original morphological descriptions, reference specimens and voucher photographs. These materials are recognised as supporting identification [191]. As these documents and supporting materials were produced by experts in the relevant taxonomic group(s), they are considered to be validated tests under the current Standard. The laboratory must have the expertise to select and justify the morphological and morphometric methods used, particularly for methods that are not described in international standards or the peer-reviewed literature. For other types of tests, the validation process must consider the technical requirements necessary to evaluate the test’s main performance characteristics. These include analytical sensitivity (the ability to detect low quantities of the target), analytical specificity (the ability to distinguish the target from non-target organisms), and selectivity. It must also evaluate repeatability (the consistency of results within the same laboratory) and reproducibility (the consistency of results between different laboratories). The guidelines reported in Tables A2–A7 of Appendix 5 of the Standard-Diagnostics EPPO PM 7/98 [191] provide detailed guidance for each parameter, depending on the method to be validated. The validation process, using the aforementioned parameters, is preceded by an initial phase of test development and optimisation. During this phase, the assay is designed with explicit diagnostic objectives. These may include diagnosing plant infections, monitoring activity, certifying pathogen-free plant material and conducting epidemiological surveys. At this stage, it is crucial to gain a thorough understanding of the pathosystem under study, encompassing the host (with regard to its resistance or susceptibility), pathogenic and virulence mechanisms, and the sampling matrix. In the case of molecular diagnostic methods, developing a diagnostic assay requires an in-depth preliminary study of the genetic diversity of the target species and its close relatives. Genome sequencing provides powerful tools for screening hypervariable genomic regions in order to detect sequence polymorphism on the basis of which to design taxon-specific oligonucleotides. Optimising experimental conditions involves determining the ideal reagent concentrations and amplification parameters and employing positive and negative controls to verify the method’s validity and efficiency. The next stage involves evaluating the performance of the test according to the above-mentioned parameters (Figure 10), namely inclusive and exclusive analytical specificity, analytical sensitivity and selectivity. The assay must demonstrate its ability to accurately discriminate between target and non-target DNA, detect and quantify minimal concentrations of target DNA, and prevent false positives in the presence of potential inhibitors (e.g., polysaccharides, tannins or phenols). Subsequently, repeatability is evaluated, which is defined as the ability of the test to provide consistent results when applied multiple times to the same sample within the same laboratory. Reproducibility, on the other hand, assesses the robustness of the methodology when identical aliquots are analyzed in different laboratories, on different days, and with different equipment when relevant [191,192].

## 8. Conclusions

Globalisation of the plant trade, driven by rising economic demands, has significantly increased the risk of *Phytophthora* spp. being introduced and spread across continents. Evidence suggests that multiple *Phytophthora* spp. often occur together in plant nurseries [31], creating ideal conditions for transmission through traded plant material. This concomitant occurrence promotes accelerated evolution, including the occurrence of hybridisation events that can lead to the emergence of new, more virulent and adaptable strains [193]. The probability that hybridization will occur increases as more infected plants are traded and planted outside their natural geographic range, and new diseases may arise as a result [194]. Controlling the movement of these pathogens through trade is a critical objective for plant health specialists. Yet achieving this goal is complicated by various challenges, such as the hidden nature of infections and the intricacy of international trade networks.

Therefore, achieving ‘pathogen-free’ status is extremely challenging. A notable example is the 2011 study by Zellner et al. [195], which revealed an average infection rate of 11% with *Phytophthora infestans* in certified European seed potatoes. The study also indicated that infection rates could reach 38%. *Phytophthora* pathogens continue to pose a significant threat to both agricultural productivity and forest ecosystems, given their capacity to infect a diverse array of hosts and cause considerable ecological and economic damage. As previously noted, traditional diagnostic methods, although indispensable, often fall short in delivering the necessary speed, sensitivity and specificity required for effective management in the context of contemporary rapid trade practices. Recent advancements in molecular diagnostics have markedly improved our ability to detect *Phytophthora* swiftly and accurately, with some methods even enabling on-site testing. Such rapid diagnostic tools are crucial not only for meeting commercial demands but also for enabling prompt interventions aimed at reducing pathogen establishment and dissemination. However, continued method development is essential to address the genetic variability and evolving populations of *Phytophthora*, as well as the challenges involved in detecting asymptomatic infections. Future research should focus on refining detection technologies to enhance specificity, reduce cross-reactivity among closely related taxa, and ensure reliability across diverse environmental samples. The integration of molecular diagnostics with risk-based screening strategies, such as targeting imports from high-risk regions or specific host species, would strengthen strategic defences against unintentional pathogen introductions. Furthermore, sustained investment in pathogen surveillance databases and international information exchange will be vital for informing quarantine protocols and guiding diagnostic priorities. Ultimately, protecting agriculture, forestry and natural ecosystems from the expanding threat posed by *Phytophthora* demands a comprehensive approach: combining advanced and rapid diagnostic technologies with well-informed regulatory policies, implementing rigorous biosecurity measures, and deepening our understanding of pathogen biology. Only through such an integrated strategy can we hope to mitigate the extensive impacts of these formidable pathogens on global plant health.

## Figures and Tables

**Table 1:** Overview of the main chemical agents employed in selective media for the isolation of *Phytophthora* spp.

Chemical Name	Microorganisms Affected	Mode of Action	References
Nystatin	The majority of fungi	Binds to ergosterol in the fungal cell membrane, forming pores that cause potassium leakage, acidification, and ultimately lead to the death of the fungus	[26,49,55,56,57,60,61]
Sodium ampicillin (Ampicillin)	Gram-positive bacteria	Prevents the synthesis of the bacterial cell wall, which leads to the rupture and destruction of bacterial cells	[26,49,54,55,56,57,61]
Rifampicin	Mycobacteria and Gram-negative bacteria	Inhibits the production of proteins in host bacteria	[26,49,54,55,56,57,61]
Pentachloro-nitrobenzene (PCNB)	A wide variety of fungi	Breaks down into CO_2_, which inhibits oxygen uptake by fungal spores, thereby killing them or preventing their germination	[49,53,54,55,61,62]
3-hydroxy 5-methyl isoxyazole (Hymexazol)	Many *Pythium* and *Mortierella* spp.	Disrupts the synthesis of RNA and DNA in fungi and oomycetes	[26,49,55,56,57,61,62]
Chloramphenicol	Gram-positive and Gram-negative bacteria	Inhibits protein synthesis by blocking the peptidyl transferase activity of the bacterial ribosome	[56]
Pimaricin	The majority of fungi	Binds to ergosterol in the fungal cell membrane, forming pores that cause potassium leakage, acidification, and ultimately lead to the death of the fungus	[52,53,54,57,61,62,63]
Metil-1-(butylcarbamoyl)-2-benzimidazole carbamate (Benomyl)	The majority of fungi	Disrupts the process of mitosis	[55,62,64]
Vancomycin	Gram-positive bacteria	Inhibits the polymerization of peptidoglycans in the bacterial cell wall	[53,62]
Penicillin	Gram-positive cocci and rods, most anaerobes, and Gram-negative bacteria	Inhibits the cross-linking of peptidoglycan within the cell wall	[52,62]
Polymyxin B	Gram-negative bacteria	Destabilizes the phospholipids and lipopolysaccharides (LPS) present	[52]

**Table 2:** List of markers for polymorphism detection in *Phytophthora* spp.

Gene/Target Region	Function/Description	Position	References
60S Ribosomal Protein L10	Conserved ribosomal protein of the 60S ribosomal subunit	Nuclear	[4,150]
Beta Tubulin	Structural protein component of microtubules	Nuclear	[4,150,151,152,153,154]
Enolase	Essential enzyme in the glycolytic pathway	Nuclear	[150]
Heat Shock Protein 90 (HSP90)	Cellular chaperone protein involved in stress response	Nuclear	[4,150,152,153,154]
Internal Transcribed Spacer (ITS) region	Internal transcribed region of rDNA; widely used for species-level identification	Nuclear	[4,150,152,153,154]
Large Subunit rRNA (28S, 5′ portion)	5′ region of the large subunit ribosomal RNA gene	Nuclear	[150]
Mitochondrial cox1 locus	Mitochondrial gene encoding cytochrome oxidase subunit 1	Mitochondrial	[4,150,152,153,154]
Mitochondrial cox2 locus	Mitochondrial gene encoding cytochrome oxidase subunit 2	Mitochondrial	[151,152,155]
Mitochondrial NADH Dehydrogenase Subunit 1 (nad1)	Mitochondrial gene encoding NADH dehydrogenase subunit 1; includes flanking regions	Mitochondrial	[153,154]
Mitochondrial NADH Dehydrogenase Subunit 9 (nad9)	Mitochondrial gene encoding NADH dehydrogenase subunit 9; includes flanking regions with mitochondrial ribosomal protein S10	Mitochondrial	[151]
Mitochondrial Ribosomal Protein S10	Mitochondrial ribosomal protein	Mitochondrial	[151,156,157]
Mitochondrial Sec-Independent Transporter Protein (ymf16)	Mitochondrial transporter protein independent of the Sec pathway	Mitochondrial	[151]
TEF1	Translation elongation factor 1	Nuclear	[4,150,152]
TigA gene fusion	Transcriptional fusion of genes encoding triose-phosphate isomerase and glyceraldehyde-3-phosphate dehydrogenase	Nuclear	[150]
Ypt1	Small GTP-binding protein of the Rab family	Nuclear	[4,158]
Mitochondrial genome region between gene Atp9 and gene Nad9	Intergenic mitochondrial DNA spacer between atp9 and nad9	Mitochondrial	[159,160,161]
Intergenic spacer (IGS) region of the rDNA	Non-transcribed region between rDNA repeats	Nuclear	[96,160]

**Table 3:** An overview of the main diagnostic protocols, along with the key features and aspects of each.

Diagnostic Assays	Protocols	Multiplex Feasibility	^4^ Efficiency	Quantification	^6^ Cost	^4^ Sensitivity	^4^ Specificity	^7^ Turnaround Time	^5^ Field- Deployability	^5^ Expertise Required	References
Traditional approaches	Cultivation- based method	N	-+	Y/N	low	+++	-	d/w	N	Y	[168,169]
	Baiting	N	-+	N	very low	++	-	d/w	Y	Y	[74,80,170]
Immunoassays	^1^ LFIA	Y	+	N	low	+	+	m	Y	N	[171]
	^1^ ELISA	N	++	Y	medium	+	+	m/h	N	Y	[172,173]
PCR	^2^ cPCR	N	++	N	medium	++	++	h	N	Y	[93,105,174,175,176,177]
	^2^ nPCR	N	+++	N	medium	++	+++	h	N	Y	
	^2^ mPCR	Y	++	N	medium	+(+)	++	h	N	Y	
	^2^ qPCR	Y/N	+++	Y	medium	+++	+++	h	N	Y	
	^2^ ddPCR	Y/N	+++	Y	high	++++	+++	h	N	Y	
Isothermal amplification	^3^ LAMP	Y/N	++	Y/N	low/medium	++	+++	m	Y	N	[178,179,180,181,182,183,184]
	^3^ RPA	Y/N	++	Y/N	low/medium	++	++	m	Y	N	
	^3^ HDA	Y/N	++	Y/N	low/medium	++	++	h	Y	N	
Hydridization	Micro- Macro arrays	Y/N	++	Y/N	high	++	+++	h	N	Y	[185]
	CRISPR-Cas	Y	++	Y/N	medium	+++	+++	m/h	Y	Y	[186]
NGS		Y	++++	Y/N	very high	++	++++	d	N	Y	[187,188]
VOCS		N	+	Y/N	low/medium	++	++	h/d	Y/N	Y	[189]

^1^ ELISA, enzyme-linked immunosorbent assay; LFIA, lateral flow immunoassay. ^2^ cPCR, conventional PCR; mPCR, multiplex PCR; nPCR, nested PCR; qPCR, quantitative PCR; ddPCR, digital droplet PCR. ^3^ LAMP, loop-mediated isothermal amplification; RPA, recombinase polymerase amplification; HDA, helicase-dependent amplification. ^4^ The number of plus signs (+) is a measure for the performance of the techniques with regard to each specific property (+ moderate; ++ good; +++ very good; ++++ excellent); - not applicable. ^5^ Indication of whether multiplexing, quantification or field deployability are possible (Y), limited (Y/N) or impossible (N). ^6^ Very low (0–10 €); low (10–60 €); medium (60–110 €); high (110–160 €); very high (>160 €). ^7^ m (minutes); h (hour(s)); d (day(s)); w (week(s)).

## Data Availability

The original contributions presented in this study are included in the article. Further inquiries can be directed to the corresponding author(s).
